# Stepwise metabolic adaption from pure metabolization to balanced anaerobic growth on xylose explored for recombinant *Saccharomyces cerevisiae*

**DOI:** 10.1186/1475-2859-13-37

**Published:** 2014-03-08

**Authors:** Mario Klimacek, Elisabeth Kirl, Stefan Krahulec, Karin Longus, Vera Novy, Bernd Nidetzky

**Affiliations:** 1 University of Technology Graz, Institute of Biotechnology and Biochemical Engineering, Petersgasse 12/I, 8010 Graz, Austria

**Keywords:** Xylose fermentation, *Saccharomyces cerevisiae*, Bio-ethanol, Evolutionary engineering, Anaerobic growth, Energy demands

## Abstract

**Background:**

To effectively convert lignocellulosic feedstocks to bio-ethanol anaerobic growth on xylose constitutes an essential trait that *Saccharomyces cerevisiae* strains normally do not adopt through the selective integration of a xylose assimilation route as the rate of ATP-formation is below energy requirements for cell maintenance (*m*_ATP_). To enable cell growth extensive evolutionary and/or elaborate rational engineering is required. However the number of available strains meeting demands for process integration are limited. In this work evolutionary engineering in just two stages coupled to strain selection under strict anaerobic conditions was carried out with BP10001 as progenitor. BP10001 is an efficient (*Y*_ethanol_ = 0.35 g/g) but slow (*q*_ethanol_ = 0.05 ± 0.01 g/g_BM_/h) xylose-metabolizing recombinant strain of *Saccharomyces cerevisiae* that expresses an optimized yeast-type xylose assimilation pathway.

**Results:**

BP10001 was adapted in 5 generations to anaerobic growth on xylose by prolonged incubation for 91 days in sealed flasks. Resultant strain IBB10A02 displayed a specific growth rate *μ* of 0.025 ± 0.002 h^-1^ but produced large amounts of glycerol and xylitol. In addition growth was strongly impaired at pH below 6.0 and in the presence of weak acids. Using sequential batch selection and IBB10A02 as basis, IBB10B05 was evolved (56 generations). IBB10B05 was capable of fast (*μ* = 0.056 ± 0.003 h^-1^; *q*_ethanol_ = 0.28 ± 0.04 g/g_BM_/h), efficient (*Y*_ethanol_ = 0.35 ± 0.02 g/g), robust and balanced fermentation of xylose. Importantly, IBB10A02 and IBB10B05 displayed a stable phenotype. Unlike BP10001 both strains displayed an unprecedented biphasic formation of glycerol and xylitol along the fermentation time. Transition from a glycerol- to a xylitol-dominated growth phase, probably controlled by CO_2_/HCO_3_^-^, was accompanied by a 2.3-fold increase of *m*_ATP_ while *Y*_ATP_ (= 87 ± 7 mmol_ATP_/g_BM_) remained unaffected. As long as glycerol constituted the main by-product energetics of anaerobic growth on xylose and glucose were almost identical.

**Conclusions:**

In just 61 generation IBB10B05, displaying ~530% improved strain fitness, was evolved from BP10001. Its excellent xylose fermentation properties under industrial relevant conditions were proven and rendered it competitive. Based on detailed analysis of growth energetics we showed that *m*_ATP_ was predominantly determined by the type of polyol formed rather than, as previously assumed, substrate-specific.

## Background

With a 82% share ethanol constitutes the most frequently used bio-fuel world-wide [1]. Current industrial processes, producing more than 86 billion liters of bio-ethanol annually, rely almost exclusively on fermentation of the sugar portions of food crops [1]. However competition with the food sector, limited farmland and insufficient greenhouse gas emission-balances demand for other more sustainable feedstock solutions. Lignocellulosic biomass represents a promising alternative with high potential in this respect as long as the complete sugar fraction predominantly made of glucose and xylose is converted into ethanol at sufficiently high rates and titers. Due to its high ethanol fermentation efficiency and enormous process robustness *Saccharomyces cerevisiae* is largely used in today’s bio-ethanol plants. However *S. cerevisiae* cannot ferment xylose without incorporating a heterologous xylose assimilation pathway in the first place. In the last two decades huge efforts have thus been made to engineer recombinant *S. cerevisiae* strains capable of efficient utilization of xylose [2-7].

To enable xylose assimilation in *S. cerevisiae* basically two routes have been addressed by genetic engineering in the past. Both pathways concentrate on the isomerization of xylose to xylulose which after phosphorylation to xylulose 5-P, catalyzed by xylulose kinase (XK), is metabolized to ethanol by reactions of the pentose phosphate (PP-) pathway and glycolysis. Isomerization of xylose may proceed in one reaction catalyzed by xylose isomerase (XI) or in two steps via xylitol catalyzed by the consecutive action of a NADPH-preferring xylose reductase (XR) and a NAD^+^-specific xylitol dehydrogenase (XDH) (Additional file 1: Figure S1).

**Figure 1:**
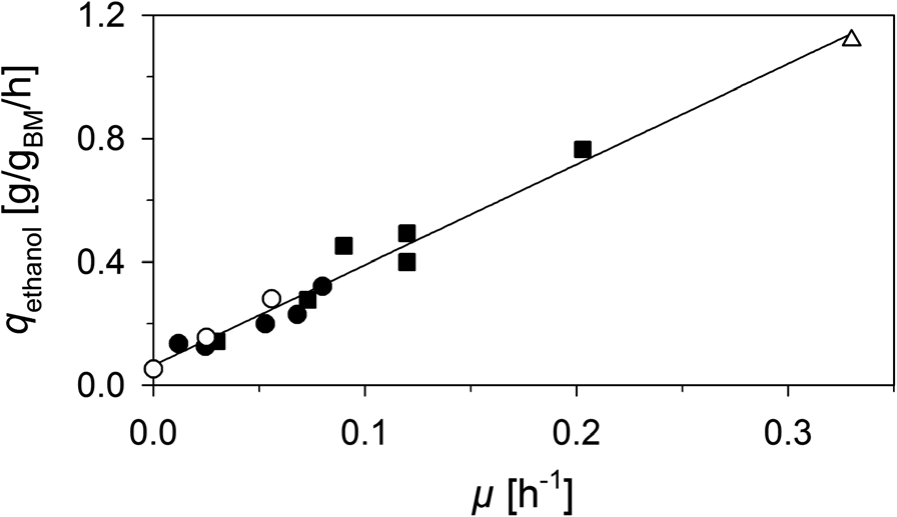
**Linear correlation between specific anaerobic growth rate of recombinant *S. cerevisiae *on xylose and specific ethanol formation rate.** This is an updated form of a graph presented previously by Almeida, J.R. and coworkers [22]. Strains expressing a XI-route or a XDH/XR- route are shown as full squares or full circles. Strains used in this study are shown as empty circles. Data for glucose fermentation (empty triangles) were from Reference [14]. Following strains were used to construct the graph (starting with the lowest *μ*): BP10001 (this study), TMB3001-C1 [8], TMB3415 [14], IBB10A02 (this study), RWB202-AFX [9], TMB3421 [15], IBB10B05 (this study), TMB3422 [15], H131-A3^SB-3^[17], TMB3420 [15], RWB17 [13], H131-A3^CS^[17], RWB18 [16], H131-A3-AL^CS^[17].

Irrespective of the route applied specific rates of ethanol formation on xylose (*q*_ethanol_ ≤ 0.05 g/g_BM_/h, where BM refers to dry cell weight) of resultant recombinant *S. cerevisiae* strains fell far below *q*_ethanol_ of glucose fermentation (~1.2 g/g_BM_/h) without further genetic modification. Evolutionary engineering [8-12] as well as rational metabolic engineering [13-15] alone or in combination [15-20] have been applied successfully to further improve *q*_ethanol_ in laboratory [8-10,13-19] and industrial strains [11,12,20]. Faster ethanol production was accompanied with the ability of these strains to grow on xylose under anaerobic conditions. Similar to anaerobic growth of *S. cerevisiae* on glucose [21], *q*_ethanol_ becomes proportional to the specific growth rate *μ* on xylose [14,22] (see Figure 1) provided that ATP needs for cell maintenance (*m*_ATP_) have been met. The onset ATP formation rate (*r*_ATP_) enabling anaerobic growth on xylose by recombinant *S. cerevisiae strains* was estimated to be 1.8 – 2.0 mmol_ATP_/g_BM_/h [23,24]. A value which would be far above maintenance requirements reported for anaerobic growth on glucose by *S. cerevisiae* (0.8 – 1.0 mmol_ATP_/g_BM_/h [21,25]) or oxygen-limited growth on xylose by *Scheffersomyces stipitis* (~1 mmol_ATP_/g_BM_/h [26]). Reasons for this large difference in *m*_ATP_ however are not known. The energy demand for growth, reflected by the slope in Figure 1, instead may be similar for both substrates.

The degree of improvement of *q*_ethanol_ in an evolutionary engineering study highly depends on the physiology of the progenitor strain used, the number of stages and generation times of the adaption process as well as the proper arrangement of enzyme activity levels potentially limiting metabolic flux. In the past years recombinant strains of *S. cerevisiae* expressing in addition to XR/XDH or XI all PP- pathway enzymes and in most cases containing a *GRE3* (encoding an unspecific NADPH-dependent aldo-keto reductase) knockout have been established within the scientific community as suitable genetic backgrounds that sufficiently facilitate high metabolic flux. Homologous expression of variants of XR from *S. stipitis* preferring NADH over NADPH together with XDH/XK or heterologous overexpression of XI together with XK in this “high flux” genetic background enabled anaerobic growth on xylose and improved ethanol productivity up to 0.32 g/g_BM_/h (XR/XDH-route [15]) and 0.45 g/g_BM_/h (XI-route [13]) even without subsequent evolutionary adaption. In combination with extensive evolutionary adaption as impressively demonstrated by Zhou H. and coworkers recombinant *S. cerevisiae* strains that fermented xylose almost as good as glucose to ethanol can be developed (see Figure 1) [17]. Although in the last years a clear trend towards XI-based strains was observable the XR/XDH-based route may have clear advantages with respect to *q*_xylose_, *q*_ethanol_ and final ethanol titer [27].

Very recently we reported on a recombinant *S. cerevisiae* strain which we have termed IBB10B05 that displayed excellent fermentation properties in spent sulfite liquor [28]. In the present work we would like to present the underlying history of strain development which was based on a very time efficient two-stage evolutionary engineering protocol. The best strains of each stage, IBB10A02 and IBB10B05, were isolated and their physiology comprehensively studied under defined medium and aeration conditions. The xylose-metabolizing strain BP10001, a recombinant of *S. cerevisiae* that homogenously expresses an optimized yeast-type xylose assimilation pathway composed of a NADH-preferring variant of *Candida tenuis* XR [29], a NAD^+^-specific XDH from *Galactocandida mastotermitis*[30] and an additional copy of endogenous XK served as genetic basis for evolutionary engineering. Construction of BP10001 [31] and comprehensive analysis with respect to physiology [31,32] and at the level of intracellular metabolites [33] have been reported elsewhere. As a result of balanced coenzyme usage of XR and XDH [33,34] BP10001 displayed efficient xylose-to-ethanol conversion capability in terms of ethanol yield (0.34 g/g). Nevertheless *q*_ethanol_ (0.05 g/g_BM_/h) was too slow to be competitive.

## Results

### Evolutionary engineering

**Figure 2:**
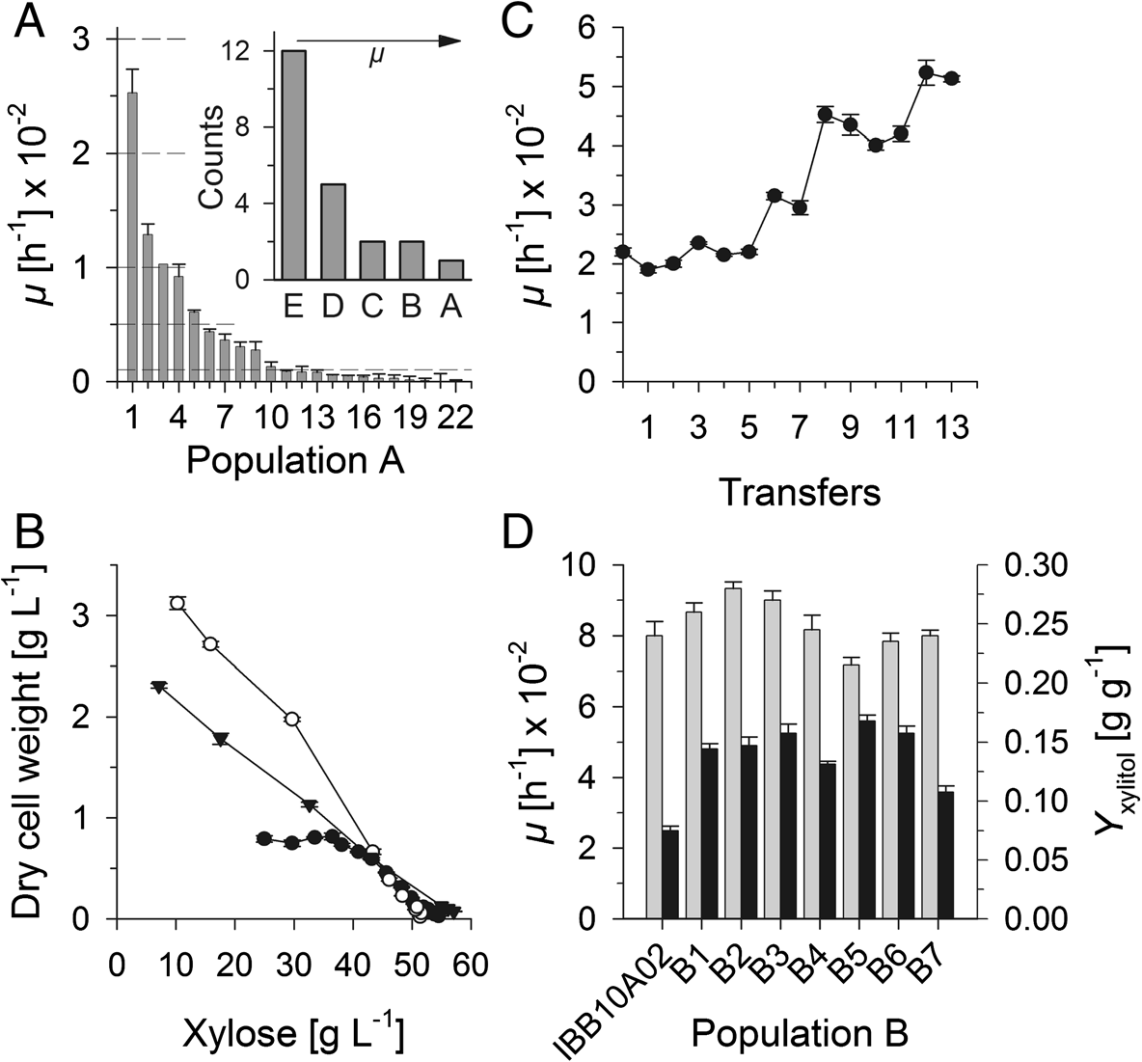
**Strain development by evolutionary engineering in two stages. Panel A**: Strains representing population A are sorted according to their *μ*. Upper bounds of classes are shown as dashed lines. Insert shows the corresponding histogram. Increase of *μ* along the classes is indicated by an arrow. **Panel B**: Growth characteristics of IBB10A02 (full circles) and IBB10B05 (empty circles) under uncontrolled and IBB10A02 under controlled (full triangles) pH conditions. **Panel C**. Evolution of population B from IBB10A02 by sequential batch selection. **Panel D**: *μ*s (black bars) and *Y*_xylitol_s (grey bars) are indicated. Error bars indicate standard deviations obtained from two individual cultivations **(Panels: A, C, D)** or from triplicate measurements of OD_600 _**(Panel B)**.

To enable anaerobic growth on xylose by BP10001 cells were incubated under anoxic conditions in defined medium containing only xylose as a carbon source for 91 days. In this time span the optical density (OD_600_) increased by a factor of 40 from OD_600_ = 0.12 to OD_600_ = 4.8, corresponding to 5.3 generations. As aerobic and anaerobic growth on xylose does not necessarily correlate [9] strain selection and screening were carried out under strict anaerobic conditions. A population of twenty two positive clones (population A) was obtained and further tested with respect to *μ*. The strain with the highest *μ* (0.025 h^-1^) was termed IBB10A02 and used for further characterization and as genetic basis in the second evolution stage. Population A which was quite heterogeneous with respect to *μ* (< 0.005 h^-1^ – 0.025 h^-1^) clustered into 5 distinct classes (see Figure 2A). The resulting strongly right-skewed distribution (inset of Figure 2A) may be therefore a direct reflection of a stepwise adaption process from BP10001-close phenotypes at the early phase of evolution to the IBB10A02 phenotype developed at a later time of experiment. Figure 2B shows a representative growth characteristic of IBB10A02 on xylose cultivated in sealed flasks. Typically cells stopped growing and started to metabolize xylose at a cell concentration of ~0.8 g_BM_/L although ~75% of xylose was still present in the medium. We observed that the pH decreased by more than 0.6 pH units in the growth phase suggesting that growth of IBB10A02 on xylose may be sensitive to pH values below 6.5. Consistent with these findings IBB10A02 was capable of growing along the entire fermentation under controlled pH (= 6.5) conditions carried out in a stirred bioreactor (see Figure 2B).

Batch-wise cultivation of IBB10A02 and serial transfer of exponentially growing cells to a new batch was carried out for sorting out faster growing populations. The enrichment process over time (transfers) is shown in Figure 2C. After 13 transfers and further 56 generations another population B was obtained with an average *μ* of 0.051 h^-1^. Seven (B1-B7) strains were selected and their strain fitness tested with respect to *μ* and *Y*_xylitol_. Results are shown in Figure 2D. Strain B5, later designated as IBB10B05, displayed the fastest growth on xylose (*μ* = 0.056 h^-1^) and lowest *Y*_xylitol_ (0.21 g/g). The *μ* of IBB10B05 was 1.8-fold higher than that of IBB10A02 (see Table 1). Furthermore IBB10B05 was capable of growth-associated utilization of xylose throughout the entire fermentation. Compared to IBB10A02 it produced about 4 times more biomass under uncontrolled pH conditions (see Figure 2B). The pH shifted by more than 1.4 pH units in this time span indicating that IBB10B05 is more robust to pH changes than IBB10A02.

**Table 1:** **Physiological parameters obtained from xylose fermentation performed under anaerobic conditions in sealed flasks at pH 6.5 together with aerobic and anaerobic specific growth rates on xylose and glucose, respectively**^**a**^

**Parameter**^**b**^	**BP10001**	**IBB10A02**		**IBB10B05**	
		**Phase 1**	**Phase 2**	**Phase 1**	**Phase 2**
*μ*_xylose_^AN^	n.d.^c^	0.025 ± 0.002		0.056 ± 0.003	
*μ*_xylose_^AE^	n.m.^c^	0.12 ± 0.01		0.16 ± 0.01	
*μ*_glucose_^AN^	0.34^d^	0.27 ± 0.02		0.26 ± 0.01	
*q*_xylose_	0.15 ± 0.04	0.50 ± 0.03		0.80 ± 0.04	
*r*_ATP_	1.1 ± 0.1	2.9 ± 0.2	3.7 ± 0.2	5.7 ± 0.4	6.3 ± 0.2
*Y*_biomass_	n.d.^c^	0.05 ± 0.01		0.07 ± 0.01	
*Y*_ethanol_	0.35 ± 0.02	0.31 ± 0.02		0.35 ± 0.02	
*Y*_xylitol_	0.19 ± 0.02	0.03 ± 0.005	0.24 ± 0.01	0.03 ± 0.01	0.20 ± 0.01
*Y*_glycerol_	0.06 ± 0.01	0.19 ± 0.02	0.015 ± 0.002	0.11 ± 0.02	0.018 ± 0.001
*Y*_acetate_	0.021 ± 0.001	0.07 ± 0.02	0.050 ± 0.002	0.04 ± 0.01	0.03 ± 0.01
*Y*_ribitol_	n.d.^c^	n.d.^c^	0.012 ± 0.003	n.d.^c^	0.011 ± 0.002
*Y*_CO2_^e^	0.35	0.35	0.33	0.37	0.36
C-Balance	0.97	1.00	1.01	0.97	1.04

^a^Concentrations of 50 g/L and 20 g/L were used for xylose and glucose, respectively; Superscripts ^AN^ and ^AE^ indicate anaerobic and aerobic cultivation conditions, respectively.

^b^*μ*, *q*_xylose_, *r*_ATP_ and *Y*_product_ are presented as h^-1^, g_xylose_/g_BM_/h, mmol_ATP_/g_BM_/h and g_product_/g_xylose_, respectively.

^c^n.d., not detectable; n.m., not measured.

^d^Data was from Ref. [33].

^e^*Y*_CO2_ was calculated based on stoichiometry of metabolic ethanol and acetate formation.

To test whether obtained phenotypes were stable, both strains were individually cultivated under conditions where growth of cells did not rely on adapted traits. To this end cells were incubated for 44 generations under aerobic conditions in complex glucose-containing medium. Five colonies of each strain were thereafter isolated and tested for their capability to ferment glucose and xylose under anaerobic conditions. Compared to corresponding *μ*s of cells not subjected to phenotype challenging conditions *μ*s obtained for each colony were not significantly affected and values for growth on glucose and xylose were identical within relative standard deviations of 3.5% (both strains) and 6.5% (both strains), respectively suggesting that phenotypes displayed by IBB10A02 and IBB10B05 are stable.

### Physiological and energetic characterization of evolved strains

**Figure 3:**
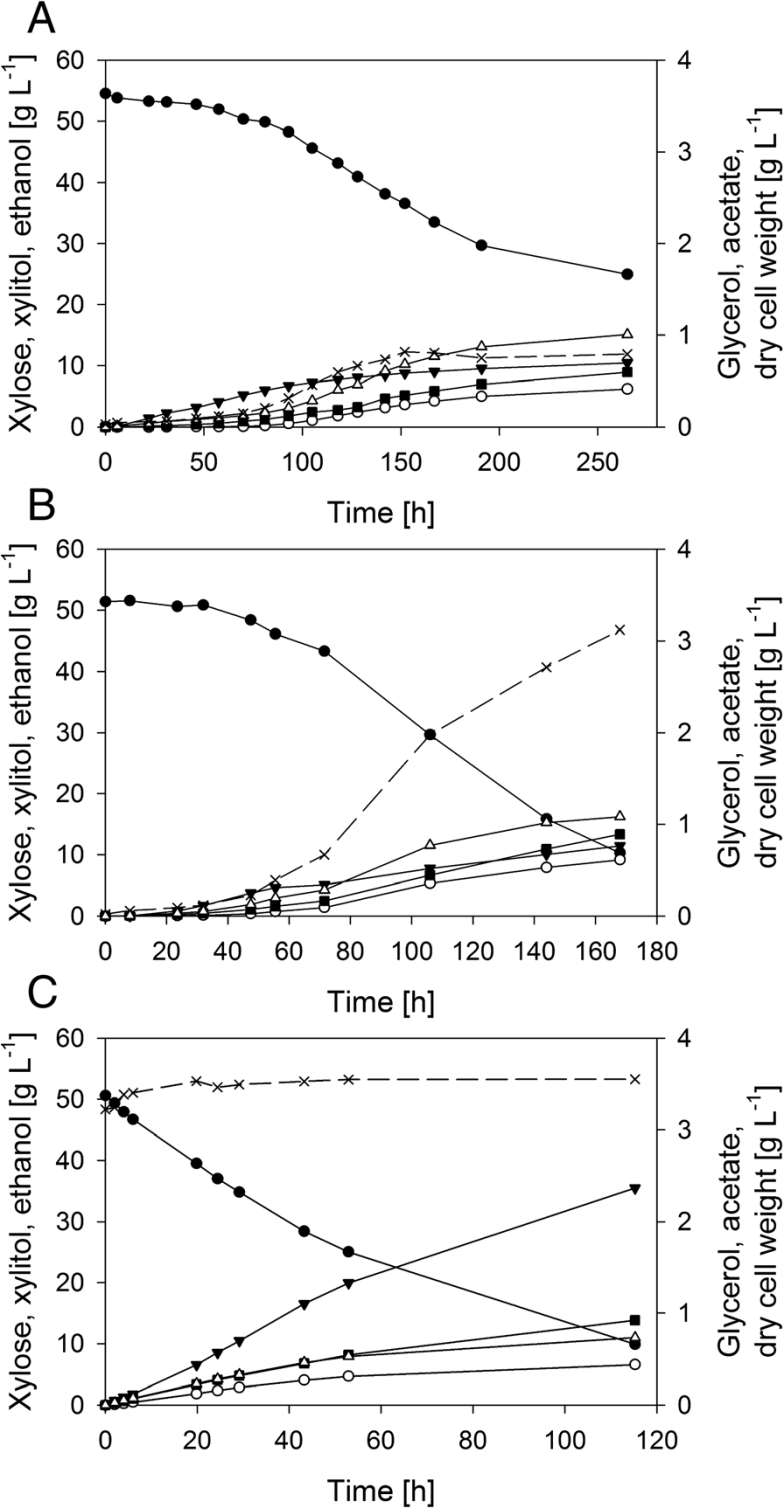
**Xylose fermentation profiles of IBB10A02 (A), IBB10B05 (B) and BP10001 (C) cultivated under anaerobic conditions in sealed flasks.** Extracellular metabolites are indicated as follows: Xylose, full circles; xylitol, empty circles; glycerol, full triangles; acetate, empty triangles; ethanol, full squares; dry cell weight, X’s linked by dashed lines.

Growth characteristics and product pattern of anaerobic xylose fermentation were studied in sealed flasks for IBB10A02 and IBB10B05 as well as for the reference strain BP10001. Representative time courses of xylose utilization and product formation are displayed in Figure 3. Resultant *μ*s, *q*_xylose_s as well as *Y*_product_s verified by respective carbon balances are summarized in Table 1. Furthermore *μ*s were determined for both evolved strains on glucose under anaerobic conditions and on xylose under aerobic conditions (see Table 1). Physiological parameters obtained for BP10001 were in excellent agreement with data reported previously for anaerobic conversion of 50 g/L xylose carried out in a stirred bioreactor at pH 5.0 [32]. IBB10A02 and IBB10B05 displayed a 3.3- and 5.3-times, respectively faster rate of xylose conversion than the reference strain. Compared to IBB10A02 IBB10B05 showed 1.3-fold faster growth on xylose under aerobic conditions while anaerobic *μ*s on glucose were almost identical for both strains and only 20% lower as compared to the corresponding *μ* of BP10001 [33]. Obtained *q*_ethanol_/*μ* pairs from xylose fermentations for both evolved strains integrated well into the relationship shown in Figure 1. Ethanol yields were high (0.35 g/g) and similar for IBB10B05 and the reference strain and only slightly lower in case of IBB10A02. Compared to their progenitor IBB10A02 and IBB10B05 produced more acetate.

**Figure 4:**
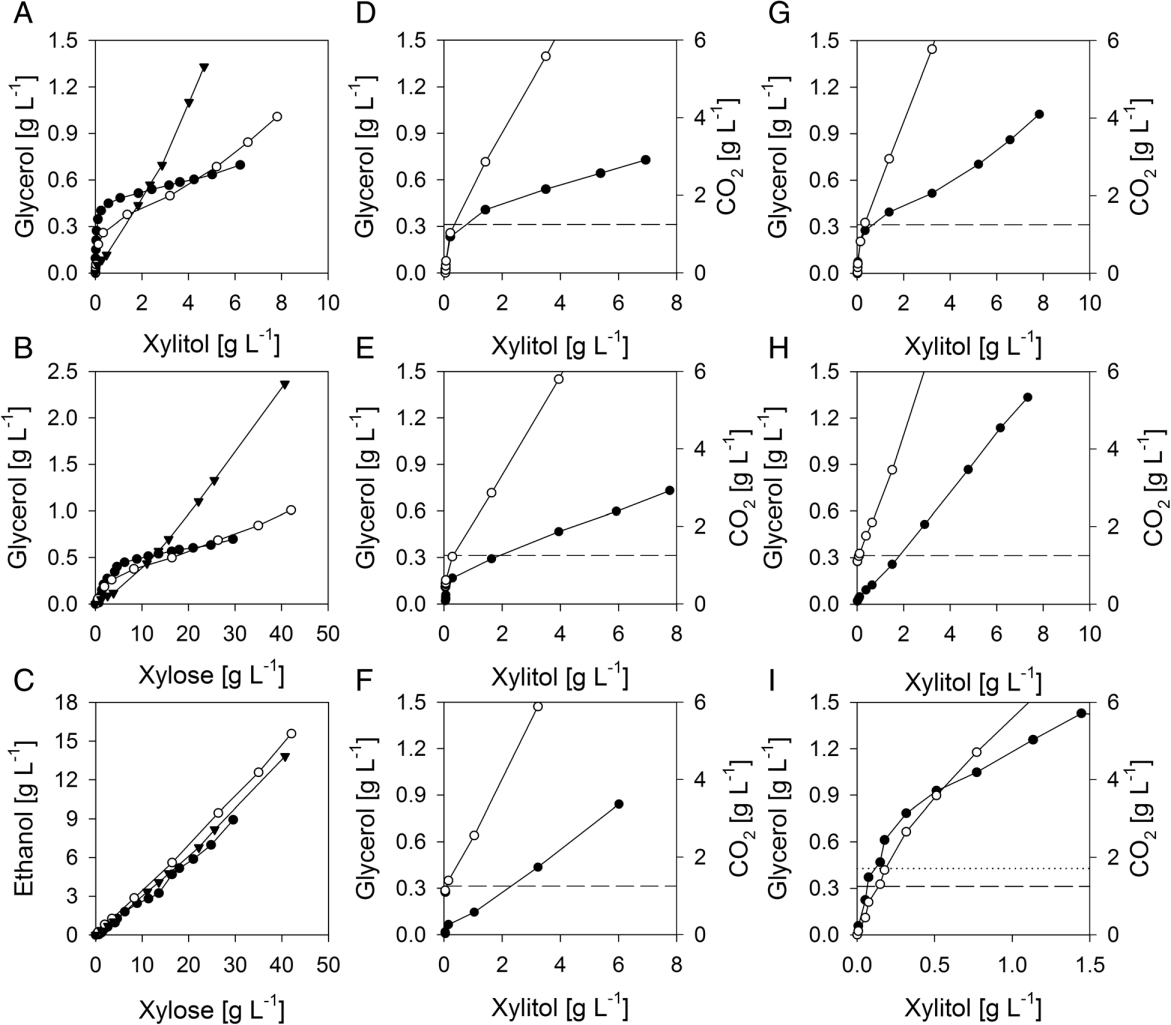
**Biphasic xylitol and glycerol production. Panels A** and **B** show glycerol formation in dependence of xylitol formation and xylose utilization, respectively. Corresponding ethanol formation is shown in **Panel C. Panels A-C**: BP10001 (full triangles), IBB10A02 (full circles), and IBB10B05 (empty circles). **Panels D-I** show glycerol (full circles) and CO_2_ (empty circles) formation in dependence of formed xylitol and added bicarbonate (initial concentrations of CO_2_ (aqueous): 0 **(Panels D and G)**; 10 mM **(Panel, E)**; 25 mM **(Panels F and H)** and fermentation form (**Panels D-H**, sealed flasks; **Panel I**, bioreactor). A p*K* of 6.5 was assumed for the relationship CO_2_ + H_2_O = H_3_O^+^ + HCO_3_^-^. Experimental data obtained for IBB10A02 and IBB10B05 are shown in **Panels D-F**, **I** and **Panels G**, **H** respectively. Dashed lines indicate saturating concentrations of CO_2_. The dotted line in **Panel I** depicts point of phase transition at 1.7 g/L CO_2_.

Unlike BP10001 both evolved strains displayed a peculiar biphasic formation characteristic for glycerol and xylitol as is portrayed in Figure 4(A, B) while *Y*_ethanol_ (Figure 4C), *Y*_biomass_ and *Y*_acetate_ were rather constant over time. In the first phase (#1) of xylose fermentation glycerol constituted the predominant by-product and only little xylitol was formed while in the second phase (#2) of fermentation the pattern switched and xylitol accumulated instead. We recognized that xylitol formation started at glycerol concentrations of 0.3 – 0.6 g/L. However, the biphasic character did not change when cultivations were performed in the presence of 0.6 g/L glycerol (data not shown). Transition from glycerol to xylitol formation was accompanied by additional release of small amounts of ribitol (~1%) a by-product not recognized in xylose-to-ethanol conversions by BP10001. Basically ribitol can be formed by *S. cerevisiae* from ribulose-5P and ribose-5P after dephosphorylation and further reduction of the resulting pentose sugars ribulose (by XDH with NADH) and ribose (by XR with NAD(P)H), respectively [35]. Because ribose constitutes a worse substrate for XR than xylose [36] and XDH can reduce ribulose with a catalytic efficiency that is 15-times faster than that for oxidizing xylitol [30,37] we may assume that predominantly XDH contributed to the formation of ribitol in both evolved strains.

ATP formation rates were calculated in accordance to Equation 1 for each strain and fermentation phase (for details see Additional file 2).

(1) $$r_{\text{ATP}}=q_{\text{ethanol}}+q_{\text{acetate}}–q_{\text{glycerol}}$$

Resultant values are shown in Table 1. Compared to BP10001 *r*_ATP_s for IBB10A02 and IBB10B05 were higher by a factor of 2.6 (#1)/3.4 (#2) and 5.2 (#1)/5.7 (#2), respectively. Energetic parameters *m*_ATP_ and *Y*_ATP_ were estimated by applying Equation 2 to *μ*s and *r*_ATP_s obtained for IBB10A02 and IBB10B05.

(2) $$r_{\text{ATP}}=Y_{\text{ATP}}*µ+m_{\text{ATP}}$$

Cells growing in the glycerol-dominated phase displayed a *m*_ATP_ of 0.7 ± 0.3 mmol_ATP_/g_BM_/h that was more than 2-fold lower than that for cells in the xylitol-dominated phase (= 1.6 ± 0.2 mmol_ATP_/g_BM_/h). Values obtained for *Y*_ATP_s were 88 ± 8 mmol_ATP_/g_BM_ (#1) and 85 ± 6 mmol_ATP_/g_BM_ (#2) and therefore not prone to the polyol produced.

### Sensitivity to pH and weak acids was dependent on the evolution stage

**Figure 5:**
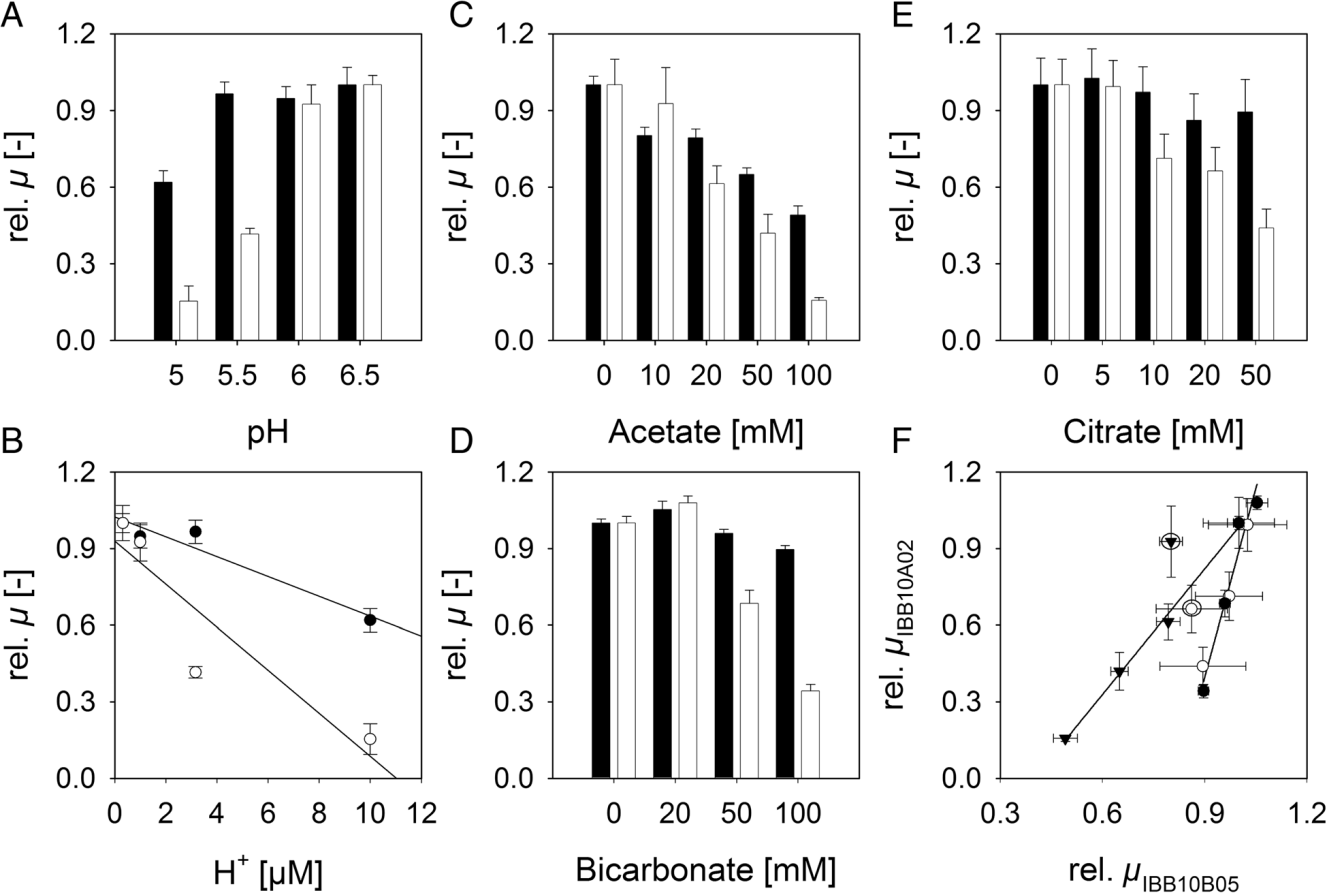
**Inhibition of anaerobic growth on xylose by pH (Panel A, B), acetate (Panel C, F), bicarbonate (Panel D, F) and citrate (Panel E, F). Panels A-E**: Experimental data obtained for IBB10A02 and IBB10B05 are shown as empty and black bars (circles), respectively. **Panel B**: x-Axis indicates concentration of protons relative to standard pH (= 6.5) conditions, ([H^+^] = 10^-pH^ – 10^-6.5^). **Panel F**: Quantitative representation of **Panels C-E**. Data obtained in the presence of acetate (full triangles), citrate (empty circles) and bicarbonate (full circles) are shown. Solid lines in **Panel B** and **F** represent best fits from linear regression analysis. Framed data were not considered in the analysis. To minimize effects on *μ* from change in pH over conversion time only the growth phase in which the pH shift was ≤ 0.5 pH units was considered in the calculations. Error bars indicate standard deviations obtained from two individual cultivations.

Evolved strains were further characterized with respect to their ability to grow at different pH values in the range of 5.0 to 6.5. Results are shown in Figure 5A. Anaerobic growth of IBB10A02 on xylose was strongly inhibited at pH values below 6.0. In contrast growth of IBB10B05 was not affected in a pH range of 5.5 – 6.5 and only weakly inhibited by ~40% at pH 5.0. Plotting concentrations of added protons versus respective relative *μ*s (see Figure 5B) showed that the inhibitory effect of protons for IBB10BA02 was ~2-times stronger than that for IBB10B05.

Weak acids constitute a significant fraction in all lignocellulosic-based hydrolysates that exerts pronounced inhibitory effects on conversion capability and growth ability of applied *S. cerevisiae* strains [22]. Three conjugate bases, acetate, citrate and bicarbonate were offered individually at different concentrations and their effects on anaerobic growth on xylose were determined for both evolved strains. Results are shown in Figure 5(C-F). Unlike *μ* of IBB10B05 which was clearly affected only by acetate within the concentration ranges tested, growth of IBB10A02 was strongly deteriorated by either acid applied. The inhibitory effect by acetate was 1.6-fold higher while those by citrate and bicarbonate were similar and ~5-fold stronger for IBB10A02 (Figure 5F).

Interestingly while addition of protons, acetate and citrate did not affect the biphasic by-product formation behavior (data not shown) increasing the amount of initial bicarbonate from 0 to 50 mM resulted in complete abolishing of the glycerol-dominated phase. Respective xylitol vs. glycerol formation plots obtained for IBB10A02 and IBB10B05 are shown in Figure 4(D-F) and (G, H), respectively. Strikingly the transition from glycerol to xylitol formation went along with CO_2_ reaching saturating concentrations (1.26 g/L [38]) in the aqueous phase. The phase transition was weaker and happened delayed with respect to CO_2_ formed when IBB10A02 was cultivated in a stirred bioreactor where the fermentation vessel was permanently purged by N_2_ and CO_2_ was steadily stripped off (Figure 4I).

### Enzyme activities

Specific enzyme activities of XR, XDH and XK were measured from cell-free extracts of BP10001, IBB10A02 and IBB10B05. Resultant values are presented in Table 2. Specific activities determined for BP10001 were in good agreement with values reported recently [39]. Enzyme activity levels were similar for both evolved strains. As a consequence of the adaption to growth on xylose activity levels of all xylose pathway enzymes were higher in evolved strains with XR levels displaying the largest increase by a factor of 12–15.

**Table 2:** **Specific enzyme activities in cell-free extracts measured at 25°C**^**a**^

**Strain**	**XR**^**b**^	**XDH**^**c**^	**XK**^**d**^
BP10001	0.08 ± 0.01	0.6 ± 0.1	1.1 ± 0.2
IBB10A02	1.0 ± 0.2	1.2 ± 0.2	1.8 ± 0.3
IBB10B05	1.2 ± 0.1	0.9 ± 0.1	1.9 ± 0.2

^a^Specific enzyme activities are presented as μmol/min/mg_protein_.

^b^Xylose, 600 mM; NADH, 0.2 mM; 100 mM potassium phosphate buffer, pH 7.0.

^c^Xylitol, 150 mM; NAD^+^, 2 mM; 100 mM Tris/HCl buffer, pH 9.0.

^d^Xylulose, 4.3 mM; NADH, 0.2 mM; ATP, 5 mM; phosphoenolpyruvate, 1 mM; bovine serum albumine, 0.88 g/L; MgCl_2_ 6H_2_O, 8.8 mM; KCl, 44 mM; 44 mM HEPES buffer, pH 7.0.

## Discussion

In this work we have used evolutionary engineering principles to enable anaerobic growth on xylose and improve ethanol productivity of BP10001, a recombinant *S. cerevisiae* strain expressing an optimized XR/XDH-route that can efficiently but slowly metabolize xylose to ethanol [31,32]. Only after 2 stages, 61 generations and 140 days and without additional rational metabolic engineering a single strain, IBB10B05, was obtained that compared to its progenitor converted xylose to ethanol at the same high yield (= 0.35 g/g) but more than 5-times faster while its ability to grow on glucose was hardly altered by the evolution process. Its xylose fermentation capability was maintained even after prolonged cultivation in complex glucose medium for more than 40 generations. Based on *q*_ethanol_ (= 0.28 g/g_BM_/h) IBB10B05 can be ranked among the top xylose converting recombinant *S. cerevisiae* strains that assimilate xylose via the XR/XDH route (see Figure 1). In this context, it is noteworthy to mention that strains displaying comparable phenotypes on xylose contained a large history of additional rational and adaptive modifications [15,17] (see Figure 1).

Evolutionary engineering presented in this work was carried out in two stages. Coherent with evolution theory [40,41] the rate of fitness gain decelerated with the number of generations (evolution stages) from 63% fitness gain per generation in the first stage to 3–4% fitness gain per generation in the second stage. The efficiency of fitness increase with which IBB10B05 evolved from BP10001 clearly surpassed the number of generations (100 – 500) typically experienced to achieve an average fitness gain of up to 50–100% [40]. Reasons for this effective adaption process may originate from (i) the relatively high *q*_ethanol_ as well as the low *Y*_xylitol_ of the ancestor BP10001, (ii) skipping aerobic and semi-aerobic evolution stages that although largely employed in this context bear the risk of selecting predominantly aerobic instead of anaerobic growing strains [9] and (iii) the selection procedure which as carried out under strict anaerobic conditions ensured selection of only those strains truly capable of anaerobic growth on xylose.

In compliance with flux control theory [42] increase of *q*_xylose_ was accompanied by an enhancement of all enzyme activity levels constituting the xylose pathway, an effect also observed by others [24]. The extent of activity level upregulation depended on the intrinsic flux capacity of pathway enzymes in the progenitor strain relative to that required to enable the new phenotype. Compared to the 3.3-fold increase of *q*_xylose_ achieved through the first evolution stage XR activity levels were increased disproportionally high (~12-fold) while those of XDH and XK were disproportionally low (~2-fold). Results therefore indicated and were in good agreement with findings from another study [33] that in particular XR activities exerted to a substantial extent flux control on *q*_xylose_ in BP10001. Further increase of *q*_xylose_ by a factor of 1.6 achieved through the second stage of adaption did not significantly affect activity levels of XR/XDH/XK indicating that other genetic modifications in the metabolism contributed to the improved phenotype of IBB10B05.

In this study we observed that predominantly the pH and the cultivation form – sealed flasks – used in the evolutionary engineering experiments contributed to a significant portion to the shaping of obtained phenotypes. Anaerobic growth on xylose evolved in the first stage was strongly inhibited by protons and weak acids which made it impossible to cultivate IBB10A02 entirely growth-associated under uncontrolled pH conditions. Xylose was, reflected by large biomass-based polyol yields (*Y*_(glycerol + xylitol)_) of 35 – 42 mmol/g_BM_, sensed by IBB10A02 as a stress compound. Observed polyol yields exceeded by far the amount of polyol typically produced by *S. cerevisiae* in the form of glycerol on glucose under anaerobic conditions (~10 mmol/g_BM_[43]) to reoxidize NADH from biomass synthesis [44]. Further improvement of *q*_ethanol_ by selection in repetitive batches not only led to faster anaerobic and aerobic growth on xylose but also enhanced acceptance of xylose as a competent fermentable substrate (*Y*_(glycerol + xylitol)_ = 20 – 22 mmol/g_BM_) as well as significantly improved resistance to pH and weak acids. In the presence of industrial relevant acetate concentrations IBB10B05 could grow at a rate ~50% of *μ* obtained under optimal conditions. Its process robustness under industrial relevant substrate conditions has been demonstrated recently [28].

Results from physiological and energetic studies provided interesting novel insights into the redox and energy metabolism of anaerobic alcohol fermentation from xylose by recombinant *S. cerevisiae*. In the first phase of xylose fermentation dominated by glycerol formation anaerobic growth on xylose was not much different from that on glucose. Energy requirements for growth (*Y*_ATP_ = 88 ± 8 mmoL_ATP_/g_BM_) and maintenance (*m*_ATP_ = 0.7 ± 0.3 mmol_ATP_/g_BM_/h) on xylose were in the same range as those typically observed for *S. cerevisiae* grown on glucose under anaerobic conditions (*Y*_ATP_ = 71 – 91 mmol_ATP_/g_BM_; *m*_ATP_ = 0.8 – 1.0 mmol_ATP_/g_BM_/h [21,25]). Remarkably almost no xylitol was formed in this fermentation phase and glycerol represented a major redox sink for regeneration of surplus NADH formed by biosynthetic processes and through acetate formation. Consequently we can assume that coenzyme recycling between XR and XDH is well balanced in both evolved strains.

Redirection of metabolic flux from glycerol to xylitol coincided with CO_2_ approaching saturating concentrations in the aqueous phase suggesting that the amount of CO_2_ or HCO_3_^-^ in the medium contributed to control of phase transition. A similar inverse relationship between CO_2_ concentrations and glycerol production, although strongly alleviated, has been reported for a wild-type strain of *S. cerevisiae*[45]. Inactivation of glycerol production however did not lead as one would have expected to an enhanced but slower ethanol production [46]. Carbon flux instead was almost quantitatively redirected towards xylitol without altering *q*_ethanol_, *μ* and *Y*_biomass_. Transition to xylitol production affected growth energetics and NADH recycling. Surplus NADH generated by biomass and acetate is now regenerated by XR. Consequently xylitol is released because coenzyme recycling between XR and XDH is no longer balanced. Energetic analysis provided evidence that energy demands of anaerobic growth on xylose for maintenance but not for growth were largely determined by the polyol, glycerol or xylitol, formed to maintain NADH balance and support osmolarity. The large *m*_ATP_ of 1.6 ± 0.3 mmol_ATP_/g_BM_/h obtained in this study for cells grown in the xylitol-dominated phase was in reasonable agreement with *m*_ATP_s of 1.8 – 2.0 mmol_ATP_/g_BM_/h suggested previously for anaerobic growth on xylose [23,24].

Interestingly based on the amount of acetate formed per biomass produced (IBB10A02: 17 – 24 mmol/g_BM_; IBB10B05: 7 – 17 mmol/g_BM_) NADPH required for biomass synthesis (6.5 mmol/g_BM_[43]) could be in principle solely supplied by acetaldehyde dehydrogenase suggesting that the oPP-pathway may play a minor role in NADPH regeneration in evolved strains. Consistent with this hypothesis Hektor and coworker found that anaerobic growth on xylose by a XI-expressing recombinant *S. cerevisiae* strain was hardly affected by inactivating the oPP-pathway [47].

## Conclusions

In this work straightforward evolution of strain fitness (530% in 61 generations) paired with low-level adaption of undesired phenotypes was achieved by carrying out all steps including selection, isolation and subsequent screening under anaerobic conditions. The resultant strain IBB10B05 displayed excellent xylose fermentation properties with respect to specific growth rate, ethanol yield and specific ethanol production rate while fermentation of glucose to ethanol was hardly affected. Its robustness under industrial relevant conditions with respect to stability of evolved phenotype, pH and weak acid concentration was proven and rendered it competitive. Adaption to balanced growth on xylose by IBB10B05 was a stepwise hierarchical process in which adaption to growth preceded metabolic adjustment to substrate and environmental conditions. We further demonstrated that the previously assumed large value of *m*_ATP_ for anaerobic growth on xylose is predominantly an effect of polyol formation rather than substrate-specific.

## Materials and methods

### Strains and cultivation conditions

The recombinant strain BP10001 (CEN.PK 113-5D ura3::(*TDH3*_p_-*XKSI*-*CYCI*_t_, *TDH3*_p_-*CtXYL1*(K274R/N276D)-*CYCI*_t_, *TDH3*_p_-*GmXYL2*-*CYCI*_t_) was used [31]. A defined mineral (M-) medium containing (NH_4_)_2_SO_4_ (5 g/L), MgSO_4_.7H_2_O (0.5 g/L), Tween-80 (0.42 mg/L), ergosterol (10 mg/L), 250 μL/L antifoam 204 (Sigma-Aldrich, Vienna, Austria), trace elements and vitamins [32,48] and K_2_HPO_4_ buffer (14.4 g/L) pH 6.5 was used. Concentrations of xylose (XM-medium) and glucose (GM-medium) were 50 g/L and 20 g/L, respectively. The pH was always adjusted prior to sterilization. All cultivations were carried out at 30°C. For long-term storage at -70°C 15% (v/v) glycerol stock cultures were prepared with cells harvested at the stationary phase and grown in baffled shake flasks on GM-medium (BP10001) or in sealed flasks on XM-medium (evolved strains).

Growth experiments under aerobic conditions were carried out at 150 rpm in 1000 mL baffled shake flasks containing 50 mL XM-medium. Initial cell density was ~0.03 g_BM_/L. Corresponding precultures were prepared in 300 mL baffled shake flasks containing 30 mL XM-medium, inoculated through the addition of 30 μL of glycerol stock culture and cultivated for 2 days. Anaerobic cultivations were carried out in 100 mL flasks (Gerresheimer Lohr GmbH, Main, Germany) sealed with a chlorobutyl rubber septum and an aluminium screw cap with 10 mm opening and containing 90 mL of X(G)M-medium. Selected agitation at 180 rpm was sufficient to prevent sedimentation of cells. Anaerobic xylose fermentation under controlled pH conditions were performed in a Labfors III bioreactor (Infors HT, Bottmingen, Switzerland) with a working volume of 2 L as described in [28]. XM-Medium used in the bioreactor contained 3 g/L K_2_HPO_4_ instead of 14.4 g/L used in flask cultivations. Experiments were initiated by the addition of biomass (BP10001: 2.5 – 3.5 g_BM_/L; evolved strains: 0.025 – 0.05 g_BM_/L). Flasks were purged for 15 min with sterile N_2_ before and after inoculation. Preparatory cultures for BP10001 and the first preculture for evolved strains were prepared by aerobic cultivation in 1000 mL and 300 mL baffled shake flasks containing GM-medium, respectively. Cultivations were started by the addition of 30 μl glycerol stock culture and incubated over night. BP10001 cells obtained were washed once with cold physiological NaCl solution prior to initiation of anaerobic xylose fermentations. Cells from evolved strains were directly transferred to sealed flasks (initial cell concentration: ~0.03 g_BM_/L) containing XM-medium or GM-medium (initiation of glucose fermentation) and further cultivated. Cells at mid-exponential phase grown in XM-medium served as inoculum for xylose fermentations.

Anaerobic growth of evolved strains was further analyzed in dependence of concentrations of hydrogen (pH 5.0 – 6.5), acetate (0 – 100 mM), citrate (0 – 50 mM), bicarbonate (0 – 100 mM) and glycerol (0.6 g/L). Each experiment was done in duplicates.

### Evolutionary engineering

Fifteen mL tubes (Pyrex^®^ Brand 9825, Fisher Scientific, Schwerte, Germany) filled with 10 mL of XM-medium were inoculated with BP10001 cells (directly from a glycerol culture) to a cell density of 0.04 g_BM_/L. Subsequently to inoculation tubes were purged with sterile N_2_ for 15 min. After 91 days of prolonged incubation at 150 rpm 400 *μ*L of cell suspension were transferred under anaerobic conditions in a Compact Glove Box 850-NB (Plas Labs Inc., MI, U.S.A.) to anaerobic cultivation (AC-) plates, containing yeast extract (8 g/L), peptone (10 g/L), xylose (20 g/L), agar-agar (13 g/L) as well as sodium thioglycolate (500 mg/L), L-cysteine (500 mg/L) and resazurin (1 mg/L), and incubated for 15 days in a 2.5 L anaerobic jar equipped with AnaeroGen bags (both Oxoid, Hampshire, England). Colonies grown were again streaked on AC-plates and incubated under exactly the same conditions for another 5 days in the anaerobic jar. Single colonies were isolated and further screened with respect to anaerobic *μ* on xylose. To this end, colonies grown on AC-plates were transferred individually to sealed flasks and cultivated as described above. The best strain obtained, with respect to *μ* (IBB10A02), was subjected to further evolutionary engineering by repetitive batches. IBB10A02 cells were therefore grown (start OD_600_ was 0.05) under anaerobic condition in sealed flasks containing XM-medium. At mid-exponential phase (OD_600_ ~ 1.0) cells were transferred to a new batch (start OD_600_ ~ 0.05) containing XM-medium and again grown until cells reached mid-exponential phase. This procedure was repeated until *μ* was approximately doubled. Positive strains were isolated and screened under anaerobic conditions.

### Analytics to cultivation experiments

Samples were withdrawn by using a hypodermic needle. OD_600_ and extracellular metabolites were analyzed spectrophotometrically at 600 nm and by HPLC equipped with a RI/UV detector [31], respectively. Concentrations of CO_2_ in the bioreactor off gas were measured with an IN1313 acoustic gas analyzer (Innova AirTechInstruments, Ballerup, Denmark) as described previously [39]. Cell dry weight to OD_600_ correlations were determined in accordance to a published protocol [31]. For BP10001 and evolved strains a correlation factor of 0.40 and 0.52 g/L dry cells per unit OD_600_ was used, respectively. The pH sensor Minitrode (Hamilton Messtechnik GmbH, Höchst, Germany) was used to measure pH in cell-free supernatants.

### Phenotype stability

Cells of IBB10A02 and IBB10B05 were cultivated in 6 repetitive batches. Cultivations were carried out under aerobic conditions in 300 mL baffled shake flasks containing 20 mL of a yeast extract (10 g/L), peptone (20 g/L), dextrose (20 g/L) (YPD) medium. Thereafter cells were streaked on YPD-agar plates and 5 colonies of each strain isolated after incubation for 48 h. Growth characteristics on glucose and xylose under anaerobic conditions of isolated colonies was analyzed.

### Enzyme activities

Specific enzyme activities were determined from cell-free extracts obtained from mid-exponentially growing cells on xylose (evolved strains) or at pseudo-steady state of xylose conversion (BP10001). Volumetric enzyme activities of XR, XDH and XK were analyzed at 25°C in accordance to [31]. Utilization or formation of NADH was recorded at 340 nm. A molar extinction coefficient of 6.22 mM^-1^ cm^-1^ was used. Protein content was determined by the Bradford method using the Roti^®^-Quant dye (Carl Roth GmbH, Karlsruhe, Germany) and bovine serum albumin as a reference.

## Abbreviations

#1: First fermentation phase; #2: Second fermentation phase; μ: Specific growth rate; AC-plates: Anaerobic cultivation plates; BM: Dry cell weight; GM-medium: M-medium containing glucose; M-medium: Mineral medium; mATP: Maintenance coefficient; OD: Optical density; oPP: Oxidative PP-pathway; PP: Pentose phosphate; q: Specific substrate conversion or product formation rate; rATP: Specific rate of ATP formation; XR: Xylose reductase; XDH: Xylitol dehydrogenase; XI: Xylose isomerase; XM-medium: M-medium containing xylose; XK: Xylulose kinase; Y: Yield; YPD: Yeast peptone dextrose.

## Competing interests

The authors declare no commercial or financial conflict of interest.

## Authors’ contributions

MK and BN designed experiments; KL performed evolutionary engineering experiments and analysis of phenotype stabilities; KL, EK, SK and VN carried out fermentation experiments and HPLC analyses; KL and EK measured specific enzyme activities; MK, EK and KL analyzed experimental data; MK and BN drafted the manuscript and MK wrote the paper; all authors have read the final version of the manuscript and given their approval.

## Supplementary Material

Additional file 1: Figure S1 Xylose assimilation routes typically employed in metabolic engineering of heterologous xylose utilization in *S. cerevisiae*. XR, XDH, XI and PP-pathway indicate xylose reductase, xylitol dehydrogenase, xylose isomerase and pentose phosphate pathway, respectively. Click here for file

Additional file 2 Estimation of ATP formation rates. Click here for file

## References

[B1] Sorda G Banse M Kemfert C An overview of biofuel policies across the world Energ Policy 2010 38 6977 6988 10.1016/j.enpol.2010.06.066

[B2] Kim SR Park YC Jin YS Seo JH Strain engineering of *Saccharomyces cerevisiae* for enhanced xylose metabolism Biotechnol Adv 2013 31 851 861 10.1016/j.biotechadv.2013.03.004 23524005

[B3] Cai Z Zhang B Li Y Engineering *Saccharomyces cerevisiae* for efficient anaerobic xylose fermentation: reflections and perspectives Biotechnol J 2012 7 34 46 10.1002/biot.201100053 22147620

[B4] Matsushika A Inoue H Kodaki T Sawayama S Ethanol production from xylose in engineered *Saccharomyces cerevisiae* strains: current state and perspectives Appl Microbiol Biotechnol 2009 84 37 53 10.1007/s00253-009-2101-x 19572128

[B5] Hahn-Hägerdal B Karhumaa K Jeppsson M Gorwa-Grauslund MF Metabolic engineering for pentose utilization in *Saccharomyces cerevisiae* Adv Biochem Eng Biotechnol 2007 108 147 177 17846723 10.1007/10_2007_062

[B6] Jeffries TW Engineering yeasts for xylose metabolism Curr Opin Biotechnol 2006 17 320 326 10.1016/j.copbio.2006.05.008 16713243

[B7] van Maris AJ Winkler AA Kuyper M de Laat WT van Dijken JP Pronk JT Development of efficient xylose fermentation in *Saccharomyces cerevisiae*: xylose isomerase as a key component Adv Biochem Eng Biotechnol 2007 108 179 204 17846724 10.1007/10_2007_057

[B8] Sonderegger M Sauer U Evolutionary engineering of *Saccharomyces cerevisiae* for anaerobic growth on xylose Appl Environ Microbiol 2003 69 1990 1998 10.1128/AEM.69.4.1990-1998.2003 12676674 PMC154834

[B9] Kuyper M Winkler AA van Dijken JP Pronk JT Minimal metabolic engineering of *Saccharomyces cerevisiae* for efficient anaerobic xylose fermentation: a proof of principle FEMS Yeast Res 2004 4 655 664 10.1016/j.femsyr.2004.01.003 15040955

[B10] Hector RE Dien BS Cotta MA Mertens JA Growth and fermentation of D-xylose by *Saccharomyces cerevisiae* expressing a novel D-xylose isomerase originating from the bacterium *Prevotella ruminicola* TC2-24 Biotechnol Biofuels 2013 6 84 10.1186/1754-6834-6-84 23721368 PMC3673840

[B11] Sonderegger M Jeppsson M Larsson C Gorwa-Grauslund MF Boles E Olsson L Spencer-Martins I Hahn-Hägerdal B Sauer U Fermentation performance of engineered and evolved xylose-fermenting *Saccharomyces cerevisiae* strains Biotechnol and Bioeng 2004 87 90 98 10.1002/bit.20094 15211492

[B12] Matsushika A Oguri E Sawayama S Evolutionary adaptation of recombinant shochu yeast for improved xylose utilization J Biosci Bioeng 2010 110 102 105 10.1016/j.jbiosc.2010.01.002 20541125

[B13] Kuyper M Hartog MM Toirkens MJ Almering MJ Winkler AA van Dijken JP Pronk JT Metabolic engineering of a xylose-isomerase-expressing *Saccharomyces cerevisiae* strain for rapid anaerobic xylose fermentation FEMS Yeast Res 2005 5 399 409 10.1016/j.femsyr.2004.09.010 15691745

[B14] Runquist D Hahn-Hägerdal B Bettiga M Increased expression of the oxidative pentose phosphate pathway and gluconeogenesis in anaerobically growing xylose-utilizing *Saccharomyces cerevisiae* Microb Cell Fact 2009 8 49 10.1186/1475-2859-8-49 19778438 PMC2760498

[B15] Runquist D Hahn-Hägerdal B Bettiga M Increased ethanol productivity in xylose-utilizing *Saccharomyces cerevisiae* via a randomly mutagenized xylose reductase Appl Environ Microbiol 2010 76 7796 7802 10.1128/AEM.01505-10 20889775 PMC2988607

[B16] Kuyper M Toirkens MJ Diderich JA Winkler AA van Dijken JP Pronk JT Evolutionary engineering of mixed-sugar utilization by a xylose-fermenting *Saccharomyces cerevisiae* strain FEMS Yeast Res 2005 5 925 934 10.1016/j.femsyr.2005.04.004 15949975

[B17] Zhou H Cheng JS Wang BL Fink GR Stephanopoulos G Xylose isomerase overexpression along with engineering of the pentose phosphate pathway and evolutionary engineering enable rapid xylose utilization and ethanol production by *Saccharomyces cerevisiae* Metab Eng 2012 14 611 622 10.1016/j.ymben.2012.07.011 22921355

[B18] Shen Y Chen X Peng B Chen L Hou J Bao X An efficient xylose-fermenting recombinant *Saccharomyces cerevisiae* strain obtained through adaptive evolution and its global transcription profile Appl Microbiol Biotechnol 2012 96 1079 1091 10.1007/s00253-012-4418-0 23053078

[B19] Peng B Shen Y Li X Chen X Hou J Bao X Improvement of xylose fermentation in respiratory-deficient xylose-fermenting *Saccharomyces cerevisiae* Metab Eng 2012 14 9 18 10.1016/j.ymben.2011.12.001 22178745

[B20] Demeke MM Dietz H Li Y Foulquié-Moreno MR Mutturi S Deprez S Den Abt T Bonini BM Liden G Dumortier F Verplaetse A Boles E Thevelein JM Development of a D-xylose fermenting and inhibitor tolerant industrial *Saccharomyces cerevisiae* strain with high performance in lignocellulose hydrolysates using metabolic and evolutionary engineering Biotechnol Biofuels 2013 6 89 10.1186/1754-6834-6-89 23800147 PMC3698012

[B21] Boender LG de Hulster EA van Maris AJ Daran-Lapujade PA Pronk JT Quantitative physiology of *Saccharomyces cerevisiae* at near-zero specific growth rates Appl Environ Microbiol 2009 75 5607 5614 10.1128/AEM.00429-09 19592533 PMC2737911

[B22] Almeida JR Runquist D Sànchez i Nogué V Lidén G Gorwa-Grauslund MF Stress-related challenges in pentose fermentation to ethanol by the yeast *Saccharomyces cerevisiae* Biotechnol J 2011 6 286 299 10.1002/biot.201000301 21305697

[B23] Wahlbom CF Hahn-Hägerdal B Furfural, 5-hydroxymethyl furfural, and acetoin act as external electron acceptors during anaerobic fermentation of xylose in recombinant *Saccharomyces cerevisiae* Biotechnol Bioeng 2002 78 172 178 10.1002/bit.10188 11870608

[B24] Sonderegger M Jeppsson M Hahn-Hägerdal B Sauer U Molecular basis for anaerobic growth of *Saccharomyces cerevisiae* on xylose, investigated by global gene expression and metabolic flux analysis Appl Environ Microbiol 2004 70 2307 2317 10.1128/AEM.70.4.2307-2317.2004 15066826 PMC383160

[B25] Verduyn C Postma E Scheffers WA van Dijken JP Energetics of *Saccharomyces cerevisiae* in anaerobic glucose-limited chemostat cultures J Gen Microbiol 1990 136 405 412 10.1099/00221287-136-3-405 2202777

[B26] Rizzi M Klein C Schulze C Bui-Thanh NA Dellweg H Xylose fermentation by yeasts. 5. use of ATP balances for modeling oxygen-limited growth and fermentation of yeast *Pichia stipitis* with xylose as carbon source Biotechnol Bioeng 1989 34 509 514 10.1002/bit.260340411 18588132

[B27] Karhumaa K Garcia Sanchez R Hahn-Hägerdal B Gorwa-Grauslund MF Comparison of the xylose reductase-xylitol dehydrogenase and the xylose isomerase pathways for xylose fermentation by recombinant *Saccharomyces cerevisiae* Microb Cell Fact 2007 6 5 10.1186/1475-2859-6-5 17280608 PMC1797182

[B28] Novy V Krahulec S Longus K Klimacek M Nidetzky B Co-fermentation of hexose and pentose sugars in a spent sulfite liquor matrix with genetically modified *Saccharomyces cerevisiae* Bioresour Technol 2013 130 439 448 23313691 10.1016/j.biortech.2012.11.115

[B29] Petschacher B Leitgeb S Kavanagh KL Wilson DK Nidetzky B The coenzyme specificity of *Candida tenuis* xylose reductase (AKR2B5) explored by site-directed mutagenesis and X-ray crystallography Biochem J 2005 385 75 83 10.1042/BJ20040363 15320875 PMC1134675

[B30] Nidetzky B Helmer H Klimacek M Lunzer R Mayer G Characterization of recombinant xylitol dehydrogenase from *Galactocandida mastotermitis* expressed in *Escherichia coli* Chem Biol Interact 2003 143–144 533 542 10.1016/s0009-2797(02)00215-6 12604239

[B31] Petschacher B Nidetzky B Altering the coenzyme preference of xylose reductase to favor utilization of NADH enhances ethanol yield from xylose in a metabolically engineered strain of *Saccharomyces cerevisiae* Microb Cell Fact 2008 7 9 10.1186/1475-2859-7-9 18346277 PMC2315639

[B32] Krahulec S Petschacher B Wallner M Longus K Klimacek M Nidetzky B Fermentation of mixed glucose-xylose substrates by engineered strains of *Saccharomyces cerevisiae*: role of the coenzyme specificity of xylose reductase, and effect of glucose on xylose utilization Microb Cell Fact 2010 9 16 10.1186/1475-2859-9-16 20219100 PMC2847541

[B33] Klimacek M Krahulec S Sauer U Nidetzky B Limitations in xylose-fermenting *Saccharomyces cerevisiae*, made evident through comprehensive metabolite profiling and thermodynamic analysis Appl Environ Microbiol 2010 76 7566 7574 10.1128/AEM.01787-10 20889786 PMC2976174

[B34] Krahulec S Klimacek M Nidetzky B Analysis and prediction of the physiological effects of altered coenzyme specificity in xylose reductase and xylitol dehydrogenase during xylose fermentation by *Saccharomyces cerevisiae* J Biotechnol 2012 158 192 202 10.1016/j.jbiotec.2011.08.026 21903144 PMC3334502

[B35] Toivari MH Ruohonen L Miasnikov AN Richard P Penttilä M Metabolic engineering of *Saccharomyces cerevisiae* for conversion of D-glucose to xylitol and other five-carbon sugars and sugar alcohols Appl Environ Microbiol 2007 73 5471 5476 10.1128/AEM.02707-06 17630301 PMC2042063

[B36] Neuhauser W Haltrich D Kulbe KD Nidetzky B Noncovalent enzyme-substrate interactions in the catalytic mechanism of yeast aldose reductase Biochemistry 1998 37 1116 1123 10.1021/bi9717800 9454604

[B37] Lunzer R Mamnun Y Haltrich D Kulbe KD Nidetzky B Structural and functional properties of a yeast xylitol dehydrogenase, a Zn^2+^-containing metalloenzyme similar to medium-chain sorbitol dehydrogenases Biochem J 1998 336 Pt 1 91 99 9806889 10.1042/bj3360091 PMC1219846

[B38] Jones RP Greenfield PF Effect of carbon dioxide on yeast growth and fermentation Enzyme Microb Tech 1982 4 210 222 10.1016/0141-0229(82)90034-5

[B39] Krahulec S Klimacek M Nidetzky B Engineering of a matched pair of xylose reductase and xylitol dehydrogenase for xylose fermentation by *Saccharomyces cerevisiae* Biotechnol J 2009 4 684 694 10.1002/biot.200800334 19452479

[B40] Dragosits M Mattanovich D Adaptive laboratory evolution – principles and applications for biotechnology Microb Cell Fact 2013 12 64 10.1186/1475-2859-12-64 23815749 PMC3716822

[B41] Sauer U Evolutionary engineering of industrially important microbial phenotypes Adv Biochem Eng Biotechnol 2001 73 129 169 11816810 10.1007/3-540-45300-8_7

[B42] Stephanopoulos G Aristidou AA Nielsen J Metabolic engineering: Principles and methodologies 1998 San Diego, London: Academic Press

[B43] Verduyn C Postma E Scheffers WA van Dijken JP Physiology of *Saccharomyces cerevisiae* in anaerobic glucose-limited chemostat cultures J Gen Microbiol 1990 136 395 403 10.1099/00221287-136-3-395 1975265

[B44] Bakker BM Overkamp KM van Maris AJ Kotter P Luttik MA van Dijken JP Pronk JT Stoichiometry and compartmentation of NADH metabolism in *Saccharomyces cerevisiae* FEMS Microbiol Rev 2001 25 15 37 10.1111/j.1574-6976.2001.tb00570.x 11152939

[B45] Aguilera J Petit T de Winde JH Pronk JT Physiological and genome-wide transcriptional responses of *Saccharomyces cerevisiae* to high carbon dioxide concentrations FEMS Yeast Res 2005 5 579 593 10.1016/j.femsyr.2004.09.009 15780657

[B46] Hubmann G Guillouet S Nevoigt E Gpd1 and Gpd2 fine-tuning for sustainable reduction of glycerol formation in *Saccharomyces cerevisiae* Appl Environ Microbiol 2011 77 5857 5867 10.1128/AEM.05338-11 21724879 PMC3165387

[B47] Hector RE Mertens JA Bowman MJ Nichols NN Cotta MA Hughes SR *Saccharomyces cerevisiae* engineered for xylose metabolism requires gluconeogenesis and the oxidative branch of the pentose phosphate pathway for aerobic xylose assimilation Yeast 2011 28 645 660 10.1002/yea.1893 21809385

[B48] Jeppsson M Bengtsson O Franke K Lee H Hahn-Hägerdal B Gorwa-Grauslund MF The expression of a *Pichia stipitis* xylose reductase mutant with higher K_M_ for NADPH increases ethanol production from xylose in recombinant *Saccharomyces cerevisiae* Biotechnol Bioeng 2006 93 665 673 10.1002/bit.20737 16372361

