# Supplementary figures and images for: Stepwise metabolic adaption from pure metabolization to balanced anaerobic growth on xylose explored for recombinant Saccharomyces cerevisiae
Source: Microb Cell Fact. 2014 Mar 8;13:37. doi: 10.1186/1475-2859-13-37 (PMC4007572; doi:10.1186/1475-2859-13-37)

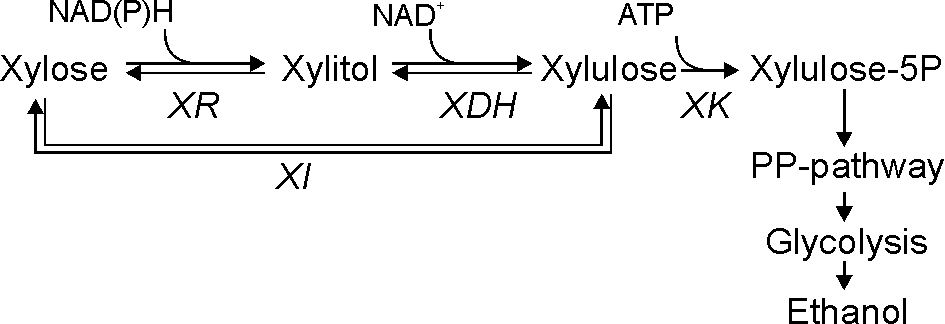


Additional File 1

Supplement: Additional file 1: Figure S1 — Xylose assimilation routes typically employed in metabolic engineering of heterologous xylose utilization in S. cerevisiae. XR, XDH, XI and PP-pathway indicate xylose reductase, xylitol dehydrogenase, xylose isomerase and pentose phosphate pathway, respectively. [file 1475-2859-13-37-S1.docx]
