# Supplementary material for: Stepwise metabolic adaption from pure metabolization to balanced anaerobic growth on xylose explored for recombinant Saccharomyces cerevisiae
Source: Microb Cell Fact. 2014 Mar 8;13:37. doi: 10.1186/1475-2859-13-37 (PMC4007572; doi:10.1186/1475-2859-13-37)

Additional File 2 to

Stepwise metabolic adaption from pure metabolization to balanced anaerobic growth on xylose explored for recombinant *Saccharomyces cerevisiae*

Klimacek Mario*, Kirl Elisabeth, Krahulec Stefan, Longus Karin, Novy Vera, Nidetzky Bernd

Estimation of ATP formation rates r_{ATP}

Equation S1 shows that for anaerobic growth on xylose by recombinant *S. cerevisiae* strains r_{ATP} can be estimated solely from the production rates of ethanol (C_2H_6O), acetate ($C_2H_4O_2$) and glycerol ($C_3H_8O_3$). Corresponding reaction equations shown by Equations S2-S6 were derived from a stoichiometric reaction network of alcohol fermentation from xylose as is illustrated in Figure S2. Two scenarios of NADPH regeneration were considered. First NADPH consumption of XR was coupled to the oPP-pathway (S2-S4) while second it was linked to acetate formation (Equations S5-S6). XR^{NADH} in Equations S2-S6 denoted the relative preference of XR for NADH (1 indicates strict NADH preference and 0 indicates strict NADPH preference).

$$r_{ATP} = q_{ethanol} + q_{acetate} - q_{glycerol} \qquad \text{(S1)}$$

Recycling of NADPH is coupled to the oPP-pathway:

$$1/6*(11XR^{NADH} - 1)*ATP + 1/6*(11XR^{NADH} - 1)*C_2H_6O + 1/3*(4XR^{NADH} + 1)*CO_2 + (1 - XR^{NADH})*C_5H_{12}O_5 - C_5H_{10}O_5 = 0 \qquad \text{(S2)}$$

$$1/3*(8 - 3XR^{NADH})*NADH + 1/3*(2 + 3XR^{NADH})*NADPH + 5/3CO_2 + 5/3C_2H_4O_2 + 5/3ATP - C_5H_{10}O_5 = 0 \qquad \text{(S3)}$$

$$1/2*(1 - XR^{NADH})*CO_2 + 1/6*(XR^{NADH} + 9)*C_3H_8O_3 - 1/6*(XR^{NADH} + 9)*ATP - 1/6*(7XR^{NADH} + 3)*NADH - C_5H_{10}O_5 = 0 \qquad \text{(S4)}$$

Recycling of NAPDH is coupled to acetate formation:

$5/3*(2XR^{NADH} - 1)*ATP + 1/3*(13XR^{NADH} - 8)*C_2H_6O + 5/3*(2XR^{NADH} - 1)*CO_2 + 2*(1-XR^{NADH})* C_5H_{12}O_5 + (1 - XR^{NADH})*C_2H_4O_2 - C_5H_{10}O_5 = 0$ (S5)

$(1/3 - 3XR^{NADH})*NADH + (1/3 - 2XR^{NADH})*ATP + (1 - XR^{NADH})*CO_2 + (XR^{NADH} + 2/3)*C_3H_8O_3 + (1 - XR^{NADH})* C_2H_4O_2 - C_5H_{10}O_5 = 0$ (S6)

Note r_{ATP} is independent of both the coenzyme usage of XR and whether NADPH regeneration is coupled to the oPP-pathway or acetate formation. In case the oPP-pathway is not active Equations S5 and S6 presented hold as long as XR^{NADH} is above 0.61. The specific rate of ethanol production represents a true estimator of r_{ATP} as long as $q_{acetate}$ and $q_{glycerol}$ are in the same range or small compared to $q_{ethanol}$.

Figure S2. Reaction network of xylose fermentation. Dual coenzyme specificity of XR was considered as well as glycerol and acetate formation. Intracellular and extracellular metabolites are shown in blue and red, respectively. Linear relationships shown in Equations S2 – S6 were obtained by metabolic flux analysis. NADH specificity of XR was varied in a range 1.0 – 0.2 (active oPP-pathway) or 1.0 – 0.61 (inactive oPP-pathway) and formation of ethanol, acetate and glycerol (active oPP-pathway) or ethanol and glycerol (inactive oPP-pathway) were used individually as objective function. Optimization of objective function was carried out by using LINDO API 5.0 (Lindo Systems Inc., Chicago, Illinois).

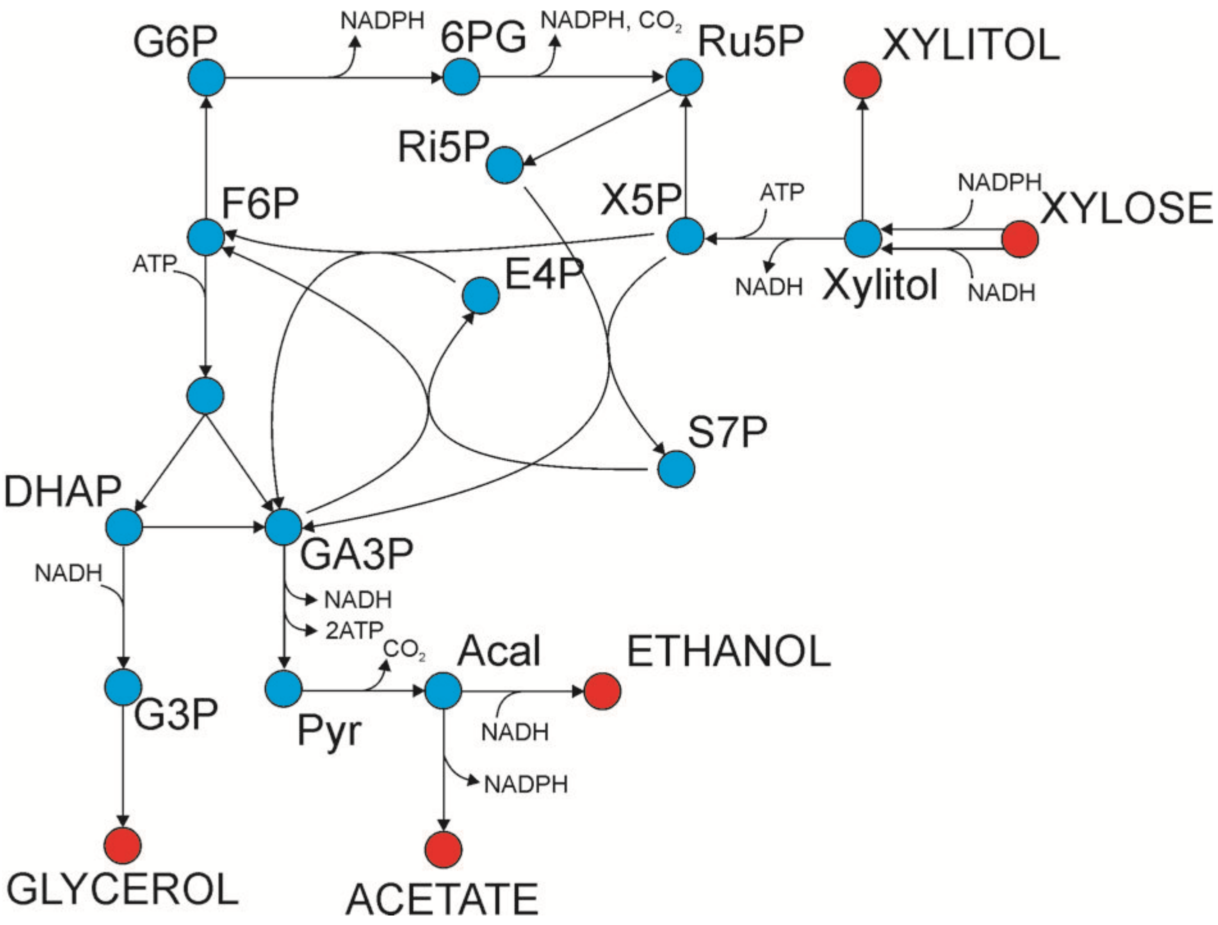

Supplement: Additional file 2 — Estimation of ATP formation rates. [file 1475-2859-13-37-S2.pdf]
